# Feeding Horses

**Published:** 1882-07

**Authors:** 


### FEEDING HORSES.

In a lecture before an English farmers' club, M. J. Storer spoke of the feeding of horses as follows :

How must horses be treated that they may be able to perform a certain amount of work without injury to their system? In the first place, they must have food; in the second place, they must have grooming; and, in the third place, they must have good stabling. In regard to food, of all animals the horse, in comparison to its size, has the smallest stomach; it is, therefore, of great importance that his food should contain as much nutriment as possible in the smallest bulk, more especially when undergoing hard work. Hay and oats have this qualification to a greater degree than any other of the feeding stuffs in general use, and that they should form the staple food has been proved by long experience. Bruised oats are very suitable for old horses and those that bolt their corn, but beyond this they have nothing specially to recommend them.

The average quantity of oats required to keep a horse undergoing hard work in good condition is about twenty pounds per day. Of course some horses would eat more ; others can not be induced to consume more than fourteen pounds. Drivers of contractors' horses are practically aware of the fact that the more they can get their horses to eat the more work they will do. But the result of over-feeding and over-working is the premature death of many valuable animals. Indian corn, when it happens to be cheap, may be advantageously used in the proportion of one to six ; the only objection to it is that it causes torpidity of the bowels. This must be counteracted by giving an equal proportion of bran. Beans, but for their heating tendency, would form a very suitable adjunct to oats, as they contain a large proportion of nutritive material. They may be safely given to animals that are hard wrought, and upward of seven years.

A horse can't be maintained in good health on grain alone ; the stomach requires a certain amount of mechanical distension to keep it properly. Hay or straw serves this purpose. Ordinary allowance should be about 20 pounds per day; something like 5

pounds in the morning, 5 pounds at midday, and 10 pounds at night. A few years ago, chopped hay became greatly in vogue; but the principal argument in its favor was that the bad hay was eaten along with the good. This tells seriously against the plan, as a horse is certainly better without bad hay in its stomach than with it.

All kinds of straw are inferior to hay, oat being the only variety that should be used; it does well when horses are idle, as they are not liable to get into too high condition on it. Green foliage is well suited to horses in its season; then the work is light, and they appear to thrive on it. It must be given in moderation, especially at first, as horses are so fond of it that they soon eat more than is good for them. Carrots, turnips, and potatoes require to be given with equal discrimination; indeed, I am inclined to condemn the use of potatoes entirely, although I have known instances where horses were allowed as many as they could eat, without bad results, but such cases are the exception, and not the rule.

Cooked food is used by many horse owners with more or less advantage, the great objection to it being that it fattens without giving strength and firmness to the muscles. It is also apt to be bolted without proper mastication, which is a common cause of colic and indigestion. For a horse recovering from any debilitating disease, or for one coming off a long journey, it is of great benefit if given judiciously. To make a regular practice of feeding with it every day, however, is unnatural, and, I believe, highly injurious. It is a common practice to give a feed of it every Saturday night for the purpose of keeping the bowels in order. Three-fourths of a pailful of mashed bran would serve the purpose better, without the risk of deranging the bowels. This is a most necessary adjunct in horse feeding, and should be given regularly once a week. It acts mechanically on the lining membrane of the stomach, increases the secretion, and thereby averts constipation.

As already stated, the stomach or receptacle for solid food is very small; the cæcum, or receptacle for water is quite the opposite. It is not uncommon to see a horse drink two or three pailfuls of water at a time. It is, therefore, probable that he does not require it often. Three times a day is sufficient, provided the horse is allowed as much as he will drink. In cases where he is excessively hot or exhausted, or where he has been kept without water for an undue length of time, it should be given in smaller quantities and more frequently. It is a great and very common error to allow horses water after being fed. In its passage through the stomach it is sure to carry with it some of the undigested food, which ought never to reach the intestines, and probably cause colic or indigestion.

